# Molecular typing reveals substantial *Plasmodium vivax* infection in asymptomatic adults in a rural area of Cameroon

**DOI:** 10.1186/1475-2875-13-170

**Published:** 2014-05-03

**Authors:** Jerome Fru-Cho, Violet V Bumah, Innocent Safeukui, Theresa Nkuo-Akenji, Vincent PK Titanji, Kasturi Haldar

**Affiliations:** 1 Department of Microbiology and Parasitology, University of Buea, Buea, South West Region, Cameroon; 2 College of Health Sciences, University of Wisconsin-Milwaukee, Milwaukee, Wisconsin, USA; 3 Center for Rare and Neglected Diseases, Department of Biological Sciences University of Notre Dame du Lac, Notre Dame, Indiana 46556, USA; 4 Biotechnology Unit, Department of Biochemistry and Molecular Biology, University of Buea, Buea, South West Region, Cameroon

**Keywords:** Asymptomatic malaria, *Plasmodium vivax*, Duffy, Anti-MSP/CSP antibody, PCR, Genotyping, Bolifamba, Cameroon

## Abstract

**Background:**

Malaria in Cameroon is due to infections by *Plasmodium falciparum* and, to a lesser extent, *Plasmodium malariae* and *Plasmodium ovale,* but rarely *Plasmodium vivax*. A recent report suggested “*Plasmodium vivax–like*” infections around the study area that remained unconfirmed. Therefore, molecular and antigenic typing was used to investigate the prevalence of *P. vivax* and Duffy in asymptomatic adults resident in Bolifamba.

**Methods:**

A cross-sectional study was conducted from July 2008 to October 2009. The status of all parasite species was determined by nested PCR in 269 blood samples collected. The *P. falciparum* and *P. vivax* anti-MSP/CSP antibody status of each subject was also determined qualitatively by a rapid card assay. Parasite DNA was extracted from a sample infected with three parasite species, purified and sequenced. The Duffy antigen status of 12 subjects infected with *P. vivax* was also determined by sequencing. *In silico* web-based tools were used to analyse sequence data for similarities and matches to reference sequences in public DNA databases.

**Results:**

The overall malaria parasite prevalence in 269 individuals was 32.3% (87) as determined by PCR. Remarkably, 14.9% (13/87) of infections were caused either exclusively or concomitantly by *P. vivax*, established both by PCR and microscopic examination of blood smears, in individuals both positive (50%, 6/12) and negative (50%, 6/12) for the Duffy receptor. A triple infection by *P. falciparum*, *P. vivax* and *P. malariae,* was detected in one infected individual*.* Anti-MSP/CSP antibodies were detected in 72.1% (194/269) of samples, indicating high and continuous exposure to infection through mosquito bites.

**Discussion:**

These data provide the first molecular evidence of *P. vivax* in Duffy positive and negative Cameroonians and suggest that there may be a significant prevalence of *P. vivax* infection than expected in the study area. Whether the *P. vivax* cases were imported or due to expansion of a founder effect was not investigated. Notwithstanding, the presence of *P. vivax* may complicate control efforts if these parasites become hypnozoitic or latent as the liver stage.

**Conclusions:**

These data strongly suggest that *P. vivax* is endemic to the south-west region of Cameroon and should be taken into account when designing malaria control strategies.

## Background

Malaria is now second to HIV/AIDS as the global cause of death from infectious diseases, but remains the main global cause of death from parasitic infectious diseases [1]. Malaria threatens approximately half the world’s population and causes debilitating illness in more than half a billion people. Morbidity and mortality is particularly high in sub-Saharan Africa, where children below five years are at greatest risk of infection, clinical disease and death [1,2]. Yet malaria control strategies have been productive: in 2010, the World Health Organization (WHO) estimated 660,000 deaths due to malaria, indicating a drop of 25% globally and 33% in the WHO African Region since 2000 [1]. *Plasmodium falciparum* remains the deadliest of the malaria parasite species in Africa [1] and also wreaks significant economic havoc in highly endemic areas, substantially decreasing gross domestic product (GDP) of affected countries relative to malaria-free regions [3,4].

With decreases in the *falciparum* burden, attention must also be focused on more than four hundred million malaria cases due to other parasite species [5], namely *Plasmodium vivax, Plasmodium malariae, Plasmodium ovale* and, more recently, *Plasmodium knowlesi* (a simian malaria parasite responsible for a zoonotic form of malaria in humans)*. Plasmodium vivax* is fast becoming a recognized cause of different grades of malaria pathologies on the African continent [6-19], infecting both children and adults, thus threatening the prospect of malaria elimination in parts of Africa. In particular, elimination of asymptomatic malaria is critical for eradication.

There have been two documented reports of infections ascribed to *P. vivax* in Cameroon [14,18]. These reports were not in subjects of African origin, but in non-Cameroonians who had lived in Cameroon previously and had returned to their home countries several years before the diagnosis indicating asymptomatic carriage. Additionally in 2005, Kimbi and colleagues [20] reported *P. vivax*-like asymptomatic infections in Cameroonian school children with a prevalence of up to 33.3%, but this was diagnosed only by light microscopy. The researchers were not very certain about speciation or species identification capabilities, and thus referred to it as “*P. vivax* -like” asymptomatic infection. This was also due to the notion that Africans for a long time were considered to be refractory to *P. vivax* infection [21], because they lack the Duffy antigen, a receptor needed for *P. vivax* to attach to and invade red blood cells. Unfortunately, there was no follow up on the “*P. vivax*-like” finding and thus there are no conclusive data on the presence of *P. vivax* infections in Cameroon.

Data on the prevalence of Duffy antigen in Cameroonians is also absent. In a bid to bridge this gap in knowledge, the present cross-sectional study was conducted with an aim to detect *Plasmodium* species and the Duffy status of asymptomatically infected adults. This group is an increasingly important pool for identifying malaria parasite species that remain prevalent in the population even as the burden of febrile (mostly *P. falciparum)* infections is systematically reduced. The participants were resident in Bolifamba, a multi-ethnic village in the South Western Cameroon rain forest zone.

## Methods

### Ethical clearance

This study was authorized by the South West Regional Delegate of Public Health and the University of Buea Institutional Review Board. All subjects gave signed informed consent before enrollment into the study. All protocols involving human subjects were approved by the IRB of the University of Notre Dame.

### Study area

The study was carried out in Bolifamba, a multi-ethnic rural setting [22] 530 m above sea level situated on the east slope of Mount Cameroon in the South West Region. Although malaria is endemic throughout Cameroon [23], the country has very different geographical and epidemiologic levels [24]. The epidemiology of malaria in Bolifamba has been well described [22]. Malaria transmission occurs all year round, with peak transmissions during the peak rainy months (July and August). There are two seasons in Bolifamba: the rainy season that runs from March to October and the dry season from November to February. The prevalence of malaria parasitaemia in this area ranges from 30% in the dry season to 65% in the rainy season [25]. *Plasmodium falciparum* accounts for up to 96% of malaria infections in this area [26], with *Anopheles gambiae s.s.* being the dominant vector [27]. Hydrologically, a stream runs through the village and is of prime importance to the villagers and to the epidemiology of the disease. In this forested area of Southern Cameroon, the equatorial climate has been modified by the double influence of the ocean and the mountain. The average humidity is constantly high, between 75-80% or more and temperatures are lower than in the other areas of the southern part of the country; the mean values of the minimum temperatures are 20°C in December and 18°C in August, the mean values of the maximum temperatures are 35°C in August and 30°C in March. Notwithstanding, seasons in Mt. Cameroon region have become quite variable in the past decade, with rains beginning in June and lasting until early November, causing some variability in malaria prevalence and transmission patterns [28]. The mountain starts at an altitude of about 50 m from the coast as a sedimentary plain that extends from Limbe to Mutengene and Tiko. From Mutengene, the terrain gradually elevates to an altitude of 800–1200 m in Buea town [29]. The mountain in Buea, as well as the Limbe Botanic garden and zoo and other recreational sites in Limbe attract tourists of various nationalities. This makes the populations along the Mount Cameroon area highly cosmopolitan. The indigenous inhabitants of Bolifamba are of several ethnic groups [22] and the main occupation is farming. Farm lands extend to the forest areas. By 2005, the population of Bolifamba was assessed to be about 3,500 inhabitants [22].

### Study group/study period

The study group consisted of apparently healthy adults resident in Bolifamba and aged 18–55 years. The subjects had lived in the area for at least five consecutive years. Subjects with signs and symptoms of any chronic illness were not included in the study.

### Sample collection

A door-to-door visit of randomly selected houses was carried out in all the quarters of the Bolifamba village. All participants were non febrile (body temperature below 37.5°C) at time of blood draw. A brief history of malaria infection and management habits was recorded for each subject. Five mL of blood was collected by venipuncture from each of the 269 subjects enrolled into EDTA containing tubes during the (peak) rainy malaria transmission months throughout the study period (July 2008 to October 2009). The blood was separated into plasma and red cell components by spinning in a Beckmann® centrifuge (Beckmann®, State Technology Inc, Bridgeport NJ, USA) at 2500 rpm for 5 min.

### Parasitological examination

Thick and thin blood smears were prepared from a small portion of whole blood of each subject on the same slide according the methods previously described [30-32]. The thin blood smears were methanol-fixed and both smears stained with Giemsa stain and examined under the oil immersion lens (x100) and x10 eye piece of an Olympus® light microscope. Parasitaemia was evaluated as previously described [31]. Essentially, with each positive smear, the level of parasitaemia was estimated by counting the parasites against at least 200 leucocytes and then using the leucocyte (WBC) count to estimate the number of parasites/μL of whole blood while assuming an average of 8,000 WBCs/μL of whole blood as shown in equation 1.

(1) $$\begin{array}{c} \text{Parasite}\;\text{Density}\;=\;\frac{\text{Number}\;\text{of}\;\text{Parasite}\;\text{Counted}}{\text{Number}\;\text{of}\;\text{WBCs}}\;\\ \;\times \;\text{WBC}\;\left(800\;\text{cells}\right)\;\text{per}\;\mathrm{\mu l}\;\text{Blood}. \end{array}$$

### Anti MSP/CSP antibody card assay

As a measure of exposure of the subjects to malaria (through infective bites of mosquitoes), the antibody status of each subject was assessed qualitatively by a rapid immunochromatographic card assay detecting either antibodies to the merozoite surface protein (MSP) or the circumsporozoite protein (CSP) (International Immuno-Diagnostics, CA, USA). The assay was carried out as described by the kit manufacturers (International Immuno-Diagnostics, CA, USA). Briefly, 10 μL of plasma sample was added to a sample well and 3 drops of the supplied assay diluent added immediately to the same sample well and then allowed to migrate by capillary action. Results were interpreted within 10–20 minutes of adding plasma and assay diluent. The appearance of a visible band corresponded to plasma reactivity with either *P. falciparum* or *P. vivax* antigens, or both. An additional control band always appeared as an internal control to enable validation of assay results and/or to ascertain that the test card was not faulty in production. When no internal control band was seen, even when the test band(s) appeared, the assay result was discarded and repeated using a fresh card.

### Nested polymerase chain reaction (PCR)

*Plasmodium* speciation was done for all the 269 subjects using the Nested Polymerase Chain Reaction (PCR) method described by Kimura *et al.,*[33] with some modifications. The Phusion® blood PCR kit was used for the Nest 1 reaction. This enabled the amplification of parasite DNA directly from 0.003% of frozen packed red cells. All PCRs were carried out in a total volume of 20 μL. The reaction mixture had final concentrations of MgCl_2_ (3.0 mM), 200 μM of each dNTP, 0.25 pmol/μL of each primer, 2.5 mM EDTA, 5% DMSO, 0.4 U Phusion Blood II DNA Polymerase. The cycling parameters for the Nest 1 reaction using the PTC-100 programmable thermal cycler (MJ. Research Inc., Waltham. MA, USA) were as follows: 1) Lysis of cells/initial denaturation 98°C for 5 minutes; 2) Denaturation at 98°C for 1 sec; 3) Annealing at 72°C for 15 sec; 4) Extension 72°C for 30 sec; 5) Repeat steps 2–4 for a total of 35 times; 6) Final annealing at 72°C for 10 minutes and 7) 4°C indefinitely.

For the Nest 2 reaction the TaKaRa kit (TaKaRa Ex Taq™, Hot Start Version) with the high fidelity HS Taq polymerase was used instead of the Phusion® blood kit. All PCRs were also carried out in a total volume of 20 μL. The reaction mixture had final concentrations of MgCl_2_ (2.005 mM), 150 μM of each dNTP, 0.25 pmol/μL of each primer, 0.05 U Taq HS DNA Polymerase. Two micro liters of a 1:50 dilution of the Nest 1 amplicon in MiliQ water (MiliQ system) was used as template in the Nest 2 reaction. The cycling parameters for the Nest 2 reaction using the PTC-100 programmable thermal cycler (MJ. Research Inc., Waltham. MA, USA) were as follows: 1) Initial denaturation 98°C for 5 minutes; 2) Denaturation at 98°C for 1 sec; 3) Annealing at 60°C for 15 sec; 4) Extension 60°C for 30 sec; 5) Repeat steps 2–4 for a total of 18 times; 6) Final annealing at 60°C for 10 minutes and 7) 4°C indefinitely. The sequence of oligonucleotides used in both the NEST 1 and 2 reactions were as previously published [6,33].

### Analysis of the amplified gene products

The Nest 2 amplicon was separated on a 2% agarose gel and a gel picture taken under UV light. All buffers and gel preparations were as described by Sambrook *et al.*[34].

### Purification and quantification of amplified DNA

The malaria parasite species bands were extracted from the gel for one subject with triple infection and purified using the QIAquick Gel Extraction Kit essentially as described by the manufacturer (Qiagen®). Five (5) μL of the purified DNA was run on a 2% agarose gel to ascertain purity and for semi-quantification.

### Sequencing/genotyping of the purified parasite DNA and DARC gene promoter DNA fragment

A sequencing order for all DNA to be sequenced was submitted (on line) to the sequencing and genotyping facility of the University of Chicago cancer center [35] and the appropriately labeled samples sent to the facility by postal mail service.

### Bioinformatics (*in silico*) analyses of the sequenced parasite DNA

The finished sequences were downloaded from the University of Chicago comprehensive cancer center web page [36], cleaned and used in a BLASTn algorithm against the GenBank reference genomic sequence of *Plasmodium* available at the NCBI web page [37] and the output file showing the hits and similarity profile copied and saved.

### Duffy blood group determination

To detect the point mutation -33 T → C, which corresponds to a Duffy-negative phenotype, the DARC gene promoter region of 13 subjects were amplified directly from frozen packed red and white cells by PCR, followed by wet lab enzymatic restriction digestion with StyI (New England Biolabs, Ipswich, MA), as well as *in silico* StyI enzymatic restriction digestion.

The PCR was done with primer pairs previously published [6] with a slight modification of the PCR constituents. The 224 bp fragment was amplified directly from diluted packed red cells or neat plasma using the Direct PCR Blood Phusion® kit (Thermo Scientific) according to the manufacturer’s instructions. All reactions were carried out in a total volume of 20 μL. The reaction mixture had final concentrations of MgCl_2_ (3.0 mM), 200 μM of each dNTP, 0.25 pmol/μL of each primer, 2.5 mM EDTA, 3% DMSO, 0.4 U Phusion Blood II DNA Polymerase and 0.5 μL of 0.001% packed red cells. The cycling parameters using the PTC-100 programmable thermal cycler (MJ. Research Inc., Waltham. MA, USA) were as follows; Lyses of cells/initial denaturation 98°C for 5 minutes, Denaturation at 98°C for 1 sec, Annealing at 59°C for 15 sec, Extension 72°C for 30 sec, Repeat steps 2–4 for a total of 35 times, final annealing at 72°C for 10 minutes and 4°C forever.

### Analysis of the amplified gene products

The amplicons were separated on a 2% agarose gel alongside a 100 bp DNA ladder and the gel photographed under UV light. All buffers and gel preparations were as described by Sambrook *et al.*[34].

### Purification and quantification of the amplified 224 bp DARC gene promoter DNA fragment

The 224 bp band corresponding to the DARC gene promoter fragment was extracted from the gel and purified using the QIAquick Gel Extraction Kit essentially as described by the manufacturer (Qiagen®). Five μL of the purified DNA was run on a 2% agarose gel to ascertain purity and for semi-quantification.

### Bioinformatics (*in silico*) analyses of the sequenced DARC gene promoter fragment

The sequences were downloaded from the University of Chicago comprehensive cancer center web page [36], cleaned and used in a multiple sequence alignment of the 12 subjects’ DARC gene promoter sequences on the NCBI web site against the DARC gene promoter Duffy positive consensus sequence downloaded from the NCBI web page. The output file showing the alignment profile was copied and saved.

### Wet lab StyI restriction digestion of the amplified DARC gene promoter fragment

In order to assess by another means whether the fragments amplified from the various subjects contained the sought T → C point mutation that confers the Duffy negative phenotype, a portion of the gel purified DNA was digested with the enzyme StyI, (New England Biolabs, NEB labs). This enzyme will cut the 224 bp fragment at 2 recognition sites to produce 3 restriction fragments of sizes 82, 77 and 64 bp, for Duffy positive genotypes or will cut at 3 recognition sites, producing 4 restriction fragments of sizes 82, 65, 64 and 12 bp, if the subject is Duffy negative. In Duffy negative subjects, the point mutation in the DARC promoter gene segment creates an additional restriction site for StyI in the 77 bp fragment, breaking it into the 65 and 12 bp fragments.

The restriction reaction was preformed according to the kit manufacturer’s instructions. Briefly, 10 μL of each purified DNA was used in the restriction mixture comprising 5 μL of miliQ water, 2 μL of 10X Bovine serum albumin (BSA), 2 μL of 10X NEBuffer 3, and 1 μL of StyI enzyme. The 20 μL total restriction volume in a 1.5 mL eppendorf tube was incubated in pre-heated water in the wells of a heating block at 37°C for at least 1 hour. The entire 20 μL tube content for each subject was mixed with 5 μL of 6x Orange G DNA loading dye (Sigma) and loaded onto a 20% DNA polyacrylamide gel (prepared as described by Sambrook *et al.*[34]), along with a 100 bp DNA ladder (Promega). The gel was run at 50 V for 3 hours. The bands were visualized under UV light on a Trans-illuminator.

### *In silico* StyI restriction digestion of the DARC promoter sequences

Given that the wet lab separation of the restriction product of the 20% native DNA PAG could be very dubious, the full DARC promoter sequence of each subject was subjected to *in silico* restriction digestion with the same enzyme (StyI) that was used in the wet lab analysis in order to confirm or refute the more convincing sequence alignment profile relative to the restriction electrophoregraph. This was done using NEBcutter 2 software freely available on the web. Each subject’s full length amplified DARC DNA sequence was interrogated with *in silico* StyI restriction enzyme and the restriction map showing number of restriction sites copied.

### Statistical analyses

The data were analysed using the Statistical Package for Social Sciences (SPSS) version 17.0 (Chicago, IL USA), and the Microsoft Excel 2010 (Microsoft Corporation). The Chi-Square test and the Pearson’s correlation were used to find correlation between categorical and numeric variables respectively. Statistical significance was set at *p* ≤ 0.05. Multiple sequence alignments were done using freely available web-based ClustalW software. The free web based NEBCutter 2 software was also available for *in silico* enzymatic restriction digestion. Sequences for analyses were either reversed or reverse complemented using free web-based softwares. Reference sequences were downloaded from respective public databases.

## Results

### Detection of Plasmodium vivax and Plasmodium falciparum infection by Giemsa

**Figure 1:**
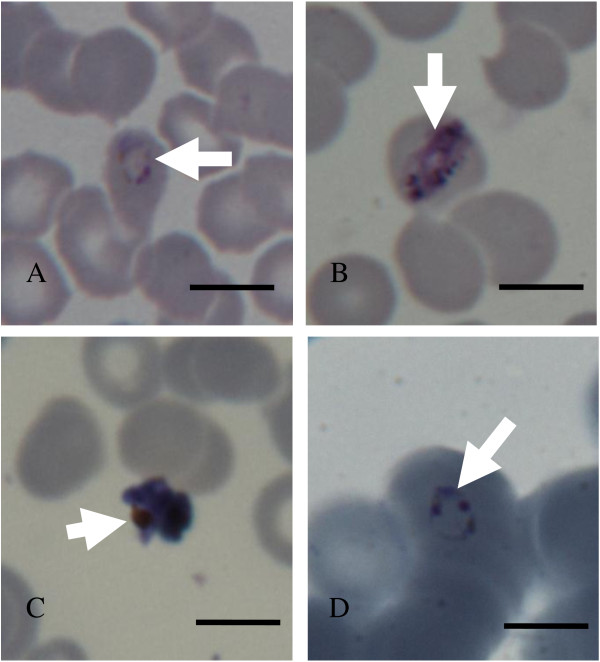
**Parasites images (arrow heads) from *Pv *positive (panels A-C) and *Pf *positive (panel D) slides.** Panel **A** shows a young trophozoite or late ring stage parasite in an intact red cell. Panel **B** shows a late stage *P. vivax* (*Pv*) trophozoite in an intact red blood cell. Panel **C** shows a schizont out of the red cell that does not look healthy. Panel **D** shows a typical *P. falciparum* (*Pf*) ring-stage parasite with double chromatin dots and pale blue cytoplasm. Scale bars = 10 μm).

Representative examples of thin films positive by Giemsa staining confirmed malarial infection suggestive of *P. falciparum* as well as other species (see Figure 1). Unfortunately, some of the morphologies shown in Figure 1 do not show hallmark characteristics of any species. Figure 1 B and C are not likely to be *P. falciparum* because they are trophozoite stage parasites and schizont stages seen in peripheral circulation. Figure 1 D does show a characteristic *P. falciparum* ring, but convincing speciation required additional analyses.

### Detection of anti-parasite antibodies to *P. vivax* and *P. falciparum* in asymptomatic subjects

To determine whether *P. vivax* was present in our samples, at first pass they were examined using antigens that will bind to both *P. vivax* and *P. falciparum* specific antibodies. This was done using qualitative anti-MSP/CSP antibody immunochromatographic card tests that distinguish between antibodies to both the MSP/CSP of *P. falciparum* and *P. vivax*. Based on this anti MSP/CSP antibody card assay, the overall prevalence of malaria exposure was 72.1% (194/269). The prevalence of single and mixed *Plasmodium* antibodies is shown on Table 1. The vast majority (66%) of samples indicated exposure to *P. falciparum* alone, ~1% exposure to *P. vivax* and about 4% exposure to both.

**Table 1:** Distribution of malarial species detected by the card test

***P. falciparum***	***P. vivax***	***P. falciparum + P. vivax***
Percent fraction of total samples shown in parenthesis*	Percent fraction of total samples shown in parenthesis*	Percent fraction of total samples shown in parenthesis*
180 (66.9)	3 (1.1)	11 (4.1)

*Total number of subjects (269).

Table 1 shows the numbers of samples (and the percent fraction of total samples) showing single, mixed infection as detected by MSP/CSP antibody card assay. A total of 269 samples were subjected to the Card test (see Methods).

### Detection of malaria species in asymptomatic subjects by nested PCR

**Figure 2:**
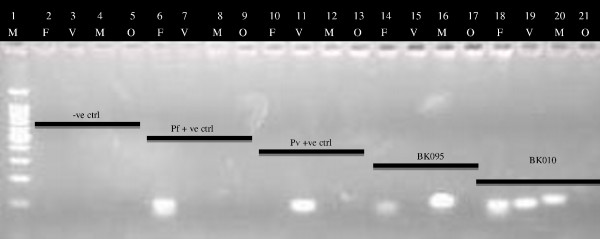
**A typical electrophoregram showing *Plasmodium *species banding patterns after the Nest 2 reaction.** In this experiment, a master mix was prepared to amplify *Plasmodium* DNA in all the samples in the Nest 1 reaction. After the Nest 2 amplification protocol, the amplicons were then separated on a 2% agarose gel (see methods for more details). M = 100 bp Ladder, F = *P. falciparum*, V = *P. vivax*, M = *P. malariae*, O = *P. ovale*. –ve ctrl = Negative control (MiliQ water), *Pf* + ve = *P. falciparum* positive control DNA, *Pv* + ve = *P. vivax* positive control DNA, BK095 & BK010 are 2 subjects’ samples.

**Figure 3:**
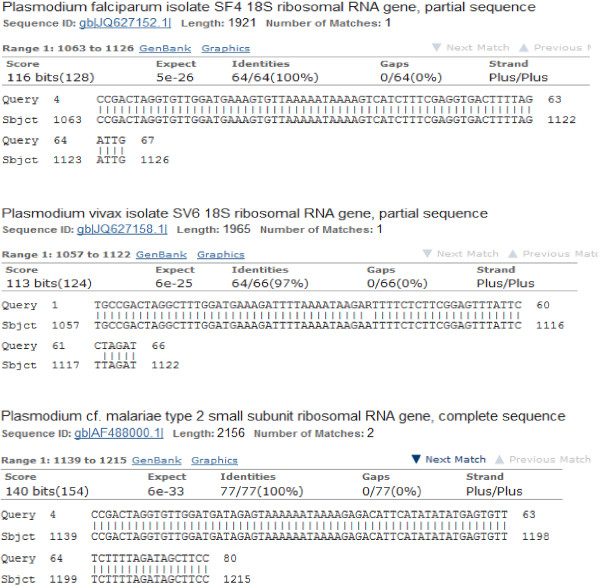
**BLASTn output of the sequences generated from the triple *Plasmodium *infected subject.** Here, each of the sequences was queried against the *Plasmodium* GeneBank data and number of hits with percentages identities displayed. For the three sequences, the Gene Bank ID of the reference sequences are shown in blue starting with “gb” for GeneBank.

Since *P. malariae* can also be found in Cameroon but not detected by the antigen kit, PCR analysis was used for definitive analysis on speciation (Figure 2). Of 269 samples collected, 66 were positive for *P. falciparum* (Table 2). Notably nine were positive for *P vivax*, four were positive for *P. malariae* and none contained *P. ovale* alone. Three were double positive *P. vivax* and *P. falciparum* another four showed both *P. malariae* and *falciparum*. One was triply positive for *P. malariae, P. falciparum* and *P. vivax* and here the primary data are shown in Figure 2. These bands were isolated and sequenced to confirm the simultaneous presence of all three species based on similarity scores (Figure 3). Although parasite morphology on the *P. vivax* positive smears (Figure 1, Panels A-C) did not all show landmark features of *P. vivax*-infected erythrocytes, the fact that they were read from slides that were solely positive for *P. vivax* by PCR was a confirmation that the parasites seen were indeed *P. vivax*. In Figure 1, Panel D shows a typical *Pf* ring from a sample that was PCR positive for just *P. falciparum*.

**Table 2:** **Mono and multiple infecting *Plasmodium *** **species in the studied subjects by PCR**

	**2008 % (n/Total n)**
***Pf***	24.5 (66/269)
***Pv***	3.3 (9/269)
***Pm***	1.5 (4/269)
***Po***	0.0 (0/269)
***Pf + Pv***	1.1 (3/269)
***Pf + Pm***	1.5 (4/269)
***Pf + Pv + Pm***	0.4 (1/269)

Pf = P. falciparum; Pv = P. vivax; Pm = P. malariae.

BLASTn analyses of the DNA sequences generated from the sample with triple bands on Figure 2 confirmed that the *P. falciparum*, *P. vivax* and *P. malariae* bands matched the Genbank *P. falciparum* isolate SF4 18S ribosomal RNA gene partial sequence ID: gb│JQ627152.1│, *P. vivax* isolate SV6 18S ribosomal RNA gene partial sequence ID: gb│JQ627158_1│, and *P. malariae* type 2 small subunit ribosomal RNA gene complete sequence ID: gb│AF488000.1│ by 100%, 97%, and 100% respectively (Figure 3).

Overall there were as many as 87 infected of a total of 269 participants, suggesting as many as a third of asymptomatic patients at this site may contain parasite reservoirs. There were many cases where the subjects were positive for parasite by PCR but negative for anti-MSP/CSP antibodies (9.3%, 25/269). This may be due to very low levels of both *P. falciparum* and *P. vivax* infection and suggests that almost a third of the *P. vivax* infections are truly asymptomatic in absence of an antibody response.

### Duffy antigen typing

**Figure 4:**
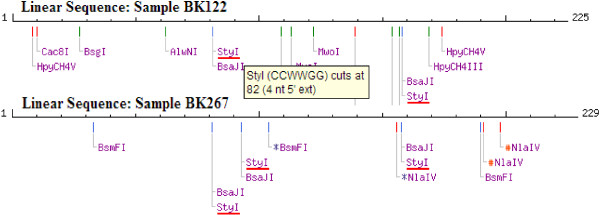
***in silico *StyI restriction map for the DARC gene promoter fragment sequence.** This shows sites for StyI restriction (red horizontal bars) in two of the subjects assessed – one Duffy positive (BK122) with 2 StyI restriction sites and the other Duffy negative (BK267) with 3 StyI restriction sites.

**Figure 5:**
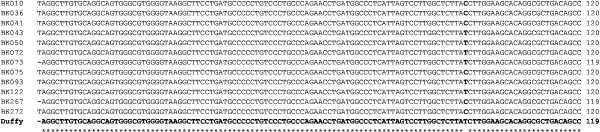
**Multiple sequence alignment of subjects’ and a Duffy positive consensus sequence, showing T → C point mutation.** The Duffy positive consensus sequence is highlighted in bold. The alignment point indicating the T → C point mutation is highlighted in bold. BK010 – BK272 represent the subject sample codes. Twelve subject samples were successfully aligned with the Duffy positive consensus sequence. Subjects BK010, BK036, BK041, BK075, BK267 and BK272 were Duffy negative, while subjects BK043, BK050, BK072, BK073, BK093 and BK122 were Duffy positive.

Having recoded a number of *P. vivax* positive cases among the study subjects by Nested PCR and confirmed by microscopy and sequenced data, the Duffy genotype of the 13 subjects with either *P. vivax* single or mixed infections was investigated. The 224 bp DARC gene promoter fragment was successfully amplified in all the samples. Each subjects’ DARC promoter DNA sequence interrogated with *in siliso* StyI restriction enzyme showed that StyI cut the Duffy positive promoter DNA sequence at two sites, producing three fragments of 82, 77 and 64 bp (Figure 4) while the Duffy negative promoter DNA sequence was cut additionally at a site created by the T → C point mutation in the 77 bp fragment, thereby producing 4 fragments of 82, 12, 65, and 64 bp (Figure 4). Multiple sequence alignment of the sequenced DARC promoter gene fragments for 12 of the 13 assessed subjects; alongside a Duffy positive consensus sequence showed the T → C point mutation which confers the Duffy negative phenotype (Figure 5). This established the prevalence of Duffy positive individuals in the present investigation at 50% (6/12). One case was lost and could not be analysed.

## Discussion

### Detection of malaria parasites in asymptomatic populations

Three methods, namely Giemsa staining, antibodies and PCR, were used to detect parasites in asymptomatic individuals. The overall prevalence of anti-parasite antibodies was relatively high (72.1%) but this largely reflected the fact that subjects were constantly exposed to infective bites of mosquitoes or sustained an immune response after active infection. Nonetheless this test and Giemsa gave the first clue that the study subjects may be infected with *P. vivax* parasites. However PCR clearly established that about a third of the study population was infected with malaria parasites. This suggests an unexpectedly high level of asymptomatic infection. Further a substantial portions of infections (~15%) contained *P. vivax*. Nonetheless this was lower than the 33.3% prevalence of microscopically diagnosed “*P. vivax*-like” infection described by Kimbi *et al*. [20]. It was surprising that *P. ovale* failed to be detected, since other reports in the area that have documented the presence of *P.ovale*[20,22,38]. As follow-up of the findings in the present study, the wider existence of *P. vivax* in Bolifamba and Cameroon should be further ascertained.

### *Plasmodium vivax* in Africa

The present report documents the first substantiated evidence of *P. vivax* infection in Cameroonian Bolifamba residents, in the SWR of Cameroon, raising the number of infecting human *Plasmodium* species in Cameroonians to four. There have been other reports of *P. vivax* elsewhere on the African continent where *P. vivax* was initially thought to be non-existent or that the population was refractory to infection. These countries include Angola [6], Equatorial Guinea [6,7], Madagascar [8], Mauritania [9], Sudan [10], Kenya [11], Sao Tomé and Principe [12-15], Congo [14], Mali [16], and Ethiopia [17]. Although the exact prevalence data for *P. vivax* in Africa is not known, the parasites seem to be more prevalent in countries where many Duffy positive people are permanent residents [8,9,11]. However, the Duffy negative status has been plausibly shown not to be a barrier to *P. vivax* infection as previously believed [8] and confirmed in other studies [6,9], as well as the current one. The increasing prevalence of *P. vivax* on the African continent and its discovery in Cameroonians could be inversely correlating to the decreasing prevalence of *P. falciparum* as a result of targeted control of the latter relative to the other malaria parasite species. A similar phenomenon has been documented in Malaysia, where an increasing incidence of *P. knowlesi* is being linked to the control of *P. falciparum* and *P. vivax*[39].

### The Duffy phenotype

With the notion that the Duffy antigen is necessary for the invasion of *P. vivax* into red cells, the Duffy genotype of all the subjects who were positive for *P. vivax* was assessed. Six (6) of the *P. vivax* infected subjects were Duffy positive (50%) and the other 6, Duffy negative. Following the three investigative molecular approaches that were used to determine the Duffy genotype of the *P. vivax*-infected individuals, two were very evident, while one was compromised by the low power of resolution of restriction fragments on a 20% native DNA PAG. The report of Duffy positive Cameroonians is a new finding. No previous study has been conducted in Cameroon to identify and determine the prevalence of the Duffy positive phenotype or genotype because of the general belief that Africans (Blacks) were largely or entirely Duffy negative and resistant to *P. vivax* infection. The findings in the present study corroborate a series of previous reports of the Duffy positive genotype on the African continent where it was thought to be non-existent and therefore a natural protecting factor for Africans against *P. vivax* infection [39,40]. Recently, Mendes and colleagues [8] showed unequivocally that the Duffy negative genotype was no longer a barrier to *P. vivax* infection, with the parasite infecting both Duffy positive and negative people, while others have implicated *P. vivax* as the causative agent in cases of severe malaria in African children [10,16].

### The locale in Cameroon

The south-west region of Cameroon is home to Mount Cameroon and there are several recreational and touristic sites that attract individuals of various nationalities. With the movement of people across geographic regions, it is likely that *P. vivax* was introduced in Cameroon a long time ago. The malaria vectors and parasites are equally changing their host range and/or adapting to new invasion strategies [29]. This is evident from the recent observation that *P. knowlesi*, originally a simian malaria species, can now infect humans [5]. A similar adaptation of the *P. vivax* parasite could be occurring, accounting for its increasing prevalence on the African continent. It is equally likely the *P. vivax* species may have been there but technology did not allow their detection. The observed prevalence of 50% for the Duffy positive antigen is relatively high and therefore cannot be ignored despite the small sample size of *P. vivax* positive subjects. The finding that both Duffy positive and negative malaria asymptomatic Bolifamba residents habored *P. vivax* parasitaemia suggests that the parasite may have evolved and could be using novel receptors to gain access into erythrocytes as proposed by some research groups [6,11,41]. This idea was most conclusively proven correct by Ménard and colleagues [8].

## Conclusions

These data provide the first definitive, molecular evidence of *P. vivax* and the Duffy positive and negative genotypes in native Bolifamba residents of Cameroon. Further investigations with a larger sample to establish the prevalence of *P. vivax* are appropriate. The results presented here indicate that there may be a need for the health sector in Cameroon to review the management of malaria to include *P. vivax.*

## Abbreviations

bp: Base pairs; BSA: Bovine serum albumin; CSP: Circumsporozoite protein; DARC: Duffy antigen chemokine receptor; DMSO: Dimethyl sulfoxide; DNA: Deoxyribonucleic acid; PAG: Polyacrylamide gel; dNTP: Deoxynucleotide triphosphate; EDTA: Ethylene diamine tetraacetate; MSP: Merozoite surface protein; Mt: Mount; NEB: New England Biolab; PCR: Polymerase chain reaction; UV: Ultra violet.

## Competing interest

The authors declare no competing financial interests.

## Authors’ contributions

JF-C was involved in all phases of the study, including study design, primary data collection and laboratory analyses of samples, statistical data analysis and interpretation, wrote-up the manuscript, and has given final approval of the version to be published. BVV designed and supervised the study, revised the manuscript and has given final approval of the version to be published. IS designed and supervised the study, analyzed and interpreted the data, revised the manuscript, and has given final approval of the version to be published. TN-A supervised the study and also revised the manuscript, and has given final approval of the version to be published. VPKT designed and supervised the study, analysed the data and also revised the manuscript, and has given final approval of the version to be published. KH designed the study, supervised the study, analysed and interpreted the data, revised the manuscript, and has given final approval of the version to be published.
